# Procalcitonin Is Not a Reliable Biomarker of Bacterial Coinfection in People With Coronavirus Disease 2019 Undergoing Microbiological Investigation at the Time of Hospital Admission

**DOI:** 10.1093/ofid/ofac179

**Published:** 2022-05-01

**Authors:** Katharine A Relph, Clark D Russell, Cameron J Fairfield, Lance Turtle, Thushan I de Silva, Matthew K Siggins, Thomas M Drake, Ryan S Thwaites, Simon Abrams, Shona C Moore, Hayley E Hardwick, Wilna Oosthuyzen, Ewen M Harrison, Annemarie B Docherty, Peter J M Openshaw, J Kenneth Baillie, Malcolm G Semple, Antonia Ho, International Severe Acute Respiratory and Emerging Infections Consortium Coronavirus Clinical Characterisation Consortium (ISARIC4C) Investigators, J Kenneth Baillie, Malcolm G Semple, Peter J M Openshaw, Gail Carson, Beatrice Alex, Benjamin Bach, Wendy S Barclay, Debby Bogaert, Meera Chand, Graham S Cooke, Annemarie B Docherty, Jake Dunning, Ana da Silva Filipe, Tom Fletcher, Christopher A Green, Ewen M Harrison, Julian A Hiscox, Antonia Ying Wai Ho, Peter W Horby, Samreen Ijaz, Saye Khoo, Paul Klenerman, Andrew Law, Wei Shen Lim, Alexander J Mentzer, Laura Merson, Alison M Meynert, Mahdad Noursadeghi, Shona C Moore, Massimo Palmarini, William A Paxton, Georgios Pollakis, Nicholas Price, Andrew Rambaut, David L Robertson, Clark D Russell, Vanessa Sancho-Shimizu, Janet T Scott, Thushan de Silva, Louise Sigfrid, Tom Solomon, Shiranee Sriskandan, David Stuart, Charlotte Summers, Richard S Tedder, Emma C Thomson, A A Roger Thompson, Ryan S Thwaites, Lance C W Turtle, Rishi K Gupta, Maria Zambon, Hayley Hardwick, Chloe Donohue, Ruth Lyons, Fiona Griffiths, Wilna Oosthuyzen, Lisa Norman, Riinu Pius, Thomas M Drake, Cameron J Fairfield, Stephen R Knight, Kenneth A Mclean, Derek Murphy, Catherine A Shaw, Jo Dalton, Michelle Girvan, Egle Saviciute, Stephanie Roberts, Janet Harrison, Laura Marsh, Marie Connor, Sophie Halpin, Clare Jackson, Carrol Gamble, Gary Leeming, Andrew Law, Murray Wham, Sara Clohisey, Ross Hendry, James Scott-Brown, William Greenhalf, Victoria Shaw, Sara McDonald, Seán Keating, Katie A Ahmed, Jane A Armstrong, Milton Ashworth, Innocent G Asiimwe, Siddharth Bakshi, Samantha L Barlow, Laura Booth, Benjamin Brennan, Katie Bullock, Benjamin W A Catterall, Jordan J Clark, Emily A Clarke, Sarah Cole, Louise Cooper, Helen Cox, Christopher Davis, Oslem Dincarslan, Chris Dunn, Philip Dyer, Angela Elliott, Anthony Evans, Lorna Finch, Lewis W S Fisher, Terry Foster, Isabel Garcia-Dorival, William Greenhalf, Philip Gunning, Catherine Hartley, Rebecca L Jensen, Christopher B Jones, Trevor R Jones, Shadia Khandaker, Katharine King, Robyn T Kiy, Chrysa Koukorava, Annette Lake, Suzannah Lant, Diane Latawiec, Lara Lavelle-Langham, Daniella Lefteri, Lauren Lett, Lucia A Livoti, Maria Mancini, Sarah McDonald, Laurence McEvoy, John McLauchlan, Soeren Metelmann, Nahida S Miah, Joanna Middleton, Joyce Mitchell, Shona C Moore, Ellen G Murphy, Rebekah Penrice-Randal, Jack Pilgrim, Tessa Prince, Will Reynolds, P Matthew Ridley, Debby Sales, Victoria E Shaw, Rebecca K Shears, Benjamin Small, Krishanthi S Subramaniam, Agnieska Szemiel, Aislynn Taggart, Jolanta Tanianis-Hughes, Jordan Thomas, Erwan Trochu, Libby van Tonder, Eve Wilcock, J Eunice Zhang, Lisa Flaherty, Nicole Maziere, Emily Cass, Alejandra Doce Carracedo, Nicola Carlucci, Anthony Holmes, Hannah Massey, Lee Murphy, Nicola Wrobel, Sarah McCafferty, Kirstie Morrice, Alan MacLean, Kayode Adeniji, Daniel Agranoff, Ken Agwuh, Dhiraj Ail, Erin L Aldera, Ana Alegria, Brian Angus, Abdul Ashish, Dougal Atkinson, Shahedal Bari, Gavin Barlow, Stella Barnass, Nicholas Barrett, Christopher Bassford, Sneha Basude, David Baxter, Michael Beadsworth, Jolanta Bernatoniene, John Berridge, Nicola Best, Pieter Bothma, David Chadwick, Robin Brittain-Long, Naomi Bulteel, Tom Burden, Andrew Burtenshaw, Vikki Caruth, David Chadwick, Duncan Chambler, Nigel Chee, Jenny Child, Srikanth Chukkambotla, Tom Clark, Paul Collini, Catherine Cosgrove, Jason Cupitt, Maria-Teresa Cutino-Moguel, Paul Dark, Chris Dawson, Samir Dervisevic, Phil Donnison, Sam Douthwaite, Ingrid DuRand, Ahilanadan Dushianthan, Tristan Dyer, Cariad Evans, Chi Eziefula, Christopher Fegan, Adam Finn, Duncan Fullerton, Sanjeev Garg, Sanjeev Garg, Atul Garg, Effrossyni Gkrania-Klotsas, Jo Godden, Arthur Goldsmith, Clive Graham, Elaine Hardy, Stuart Hartshorn, Daniel Harvey, Peter Havalda, Daniel B Hawcutt, Maria Hobrok, Luke Hodgson, Anil Hormis, Michael Jacobs, Susan Jain, Paul Jennings, Agilan Kaliappan, Vidya Kasipandian, Stephen Kegg, Michael Kelsey, Jason Kendall, Caroline Kerrison, Ian Kerslake, Oliver Koch, Gouri Koduri, George Koshy, Shondipon Laha, Steven Laird, Susan Larkin, Tamas Leiner, Patrick Lillie, James Limb, Vanessa Linnett, Jeff Little, Mark Lyttle, Michael MacMahon, Emily MacNaughton, Ravish Mankregod, Huw Masson, Elijah Matovu, Katherine McCullough, Ruth McEwen, Manjula Meda, Gary Mills, Jane Minton, Mariyam Mirfenderesky, Kavya Mohandas, Quen Mok, James Moon, Elinoor Moore, Patrick Morgan, Craig Morris, Katherine Mortimore, Samuel Moses, Mbiye Mpenge, Rohinton Mulla, Michael Murphy, Megan Nagel, Thapas Nagarajan, Mark Nelson, Matthew K O’Shea, Igor Otahal, Marlies Ostermann, Mark Pais, Selva Panchatsharam, Danai Papakonstantinou, Hassan Paraiso, Brij Patel, Natalie Pattison, Justin Pepperell, Mark Peters, Mandeep Phull, Stefania Pintus, Jagtur Singh Pooni, Frank Post, David Price, Rachel Prout, Nikolas Rae, Henrik Reschreiter, Tim Reynolds, Neil Richardson, Mark Roberts, Devender Roberts, Alistair Rose, Guy Rousseau, Brendan Ryan, Taranprit Saluja, Aarti Shah, Prad Shanmuga, Anil Sharma, Anna Shawcross, Jeremy Sizer, Manu Shankar-Hari, Richard Smith, Catherine Snelson, Nick Spittle, Nikki Staines, Tom Stambach, Richard Stewart, Pradeep Subudhi, Tamas Szakmany, Kate Tatham, Jo Thomas, Chris Thompson, Robert Thompson, Ascanio Tridente, Darell Tupper-Carey, Mary Twagira, Andrew Ustianowski, Nick Vallotton, Lisa Vincent-Smith, Shico Visuvanathan, Alan Vuylsteke, Sam Waddy, Rachel Wake, Andrew Walden, Ingeborg Welters, Tony Whitehouse, Paul Whittaker, Ashley Whittington, Padmasayee Papineni, Meme Wijesinghe, Martin Williams, Lawrence Wilson, Sarah Cole, Stephen Winchester, Martin Wiselka, Adam Wolverson, Daniel G Wootton, Andrew Workman, Bryan Yates, Peter Young

**Affiliations:** 1 University of Edinburgh Centre for Inflammation Research, Edinburgh, United Kingdom; 2 Centre for Medical Informatics, Usher Institute, University of Edinburgh, Edinburgh, United Kingdom; 3 National Institute for Health Research Health Protection Research Unit in Emerging and Zoonotic Infections, Institute of Infection, Veterinary and Ecological Sciences, Faculty of Health and Life Sciences, University of Liverpool, Liverpool, United Kingdom; 4 Liverpool University Hospitals NHS Foundation Trust, Liverpool, United Kingdom; 5 South Yorkshire Regional Department of Infection and Tropical Medicine, Sheffield Teaching Hospitals National Health Service Foundation Trust, Sheffield, United Kingdom; 6 Department of Infection, Immunity and Cardiovascular Disease, Medical School, University of Sheffield, Sheffield, United Kingdom; 7 National Heart and Lung Institute, Imperial College London, London, United Kingdom; 8 Department of Clinical Infection, Microbiology and Immunology, University of Liverpool, Liverpool, United Kingdom; 9 Division of Genetics and Genomics, Roslin Institute, University of Edinburgh, Edinburgh, United Kingdom; 10 Medical Research Council Human Genetics Unit, Institute for Genetics and Molecular Medicine, University of Edinburgh, Edinburgh, United Kingdom; 11 Intensive Care Unit, Royal Infirmary of Edinburgh, Edinburgh, United Kingdom; 12 Department of Respiratory Medicine, Alder Hey Children’s Hospital, Liverpool, United Kingdom; 13 Medical Research Council–University of Glasgow Centre for Virus Research, Glasgow, United Kingdom

**Keywords:** COVID-19, SARS-CoV-2, procalcitonin, coinfection

## Abstract

Admission procalcitonin measurements and microbiology results were available for 1040 hospitalized adults with coronavirus disease 2019 (from 48 902 included in the International Severe Acute Respiratory and Emerging Infections Consortium World Health Organization Clinical Characterisation Protocol UK study). Although procalcitonin was higher in bacterial coinfection, this was neither clinically significant (median [IQR], 0.33 [0.11–1.70] ng/mL vs 0.24 [0.10–0.90] ng/mL) nor diagnostically useful (area under the receiver operating characteristic curve, 0.56 [95% confidence interval, .51–.60]).

Antimicrobial therapy is not recommended in coronavirus disease 2019 (COVID-19) in the absence of suspected bacterial infection [1]. Meta-analyses of observational data have found that 4.9% of people with COVID-19 present with bacterial coinfection, yet around 75% receive antimicrobials [2, 3].

Procalcitonin production occurs in response to lipopolysaccharide, bacterial infection, and cytokines including interleukin 6 (IL-6) and tumor necrosis factor alpha (TNF-α) [4]. Elevated procalcitonin is used as a biomarker of bacterial infection. In acute respiratory infections, procalcitonin-guided antimicrobial usage can reduce antimicrobial exposure [5]. A substantial increase in this practice has been reported during the COVID-19 pandemic [6]. However, in a cohort of hospitalized people with COVID-19 we have previously reported a stepwise increase in procalcitonin with increasing disease severity [7].

Dysregulated innate immune responses occur in COVID-19 involving a central role for IL-6 [7]. We hypothesized that this could reduce the utility of procalcitonin as a biomarker of bacterial infection. To address this, we aimed to determine whether admission procalcitonin was associated with bacterial coinfection in hospitalized people with COVID-19 undergoing microbiological investigation.

## METHODS

### Study Design

The International Severe Acute Respiratory and Emerging Infections Consortium (ISARIC) World Health Organization (WHO) Clinical Characterisation Protocol United Kingdom (CCP-UK) study is an ongoing prospective cohort study recruiting inpatients in 260 hospitals in England, Scotland, and Wales (National Institute for Health Research Clinical Research Network Central Portfolio Management System ID: 14152) performed by the ISARIC Coronavirus Clinical Characterisation Consortium (ISARIC4C). The study protocol is available online (isaric4c.net/protocols). Patients with confirmed or clinician-defined high likelihood of severe acute respiratory syndrome coronavirus 2 (SARS-CoV-2) infection were eligible for inclusion. Ethical approval was given by the South Central–Oxford C Research Ethics Committee in England (13/SC/0149), the Scotland A Research Ethics Committee (20/SS/0028), and the WHO Ethics Review Committee (RPC571 and RPC572).

### Inclusion Criteria for Procalcitonin Analysis

We have reported microbiological findings from 48 902 patients included in the CCP-UK study, hospitalized between 6 February and 8 June 2020, with reverse-transcription polymerase chain reaction (PCR)–confirmed SARS-CoV-2 infection and an outcome recorded 28 days after admission [8]. To evaluate the utility of procalcitonin, we retrospectively analyzed a subgroup of this cohort with (1) results of a blood or respiratory culture recorded within 2 days of admission (our previous definition of “coinfection”); (2) a procalcitonin result recorded within 24 hours of admission; and (3) no positive cultures from other sample types in absence of positive blood/respiratory samples. Details of microbiology data processing are included in our previous report [8]. In brief, samples were considered negative if there was no growth or growth suggestive of contamination or colonization (eg, coagulase-negative staphylococci excluding *Streptococcus lugdunensis*, *Corynebacterium* species, or *Cutibacterium* species in blood cultures or *Candida* species in sputum). Samples positive for fungi alone were considered negative for this analysis.

### Statistical Analysis

Planned comparisons of procalcitonin values between groups were done using Mann-Whitney tests (Shapiro-Wilk normality test demonstrated a nonnormal distribution). Statistical analyses were performed using GraphPad Prism (version 9.1.2). Relationships between procalcitonin and inflammatory markers (white cell counts, C-reactive protein [CRP], IL-6, TNF-α) were assessed by correlation matrix analysis (using a Spearman test in the *corrplot* R package) or simple linear regression with correlation assessed using a Spearman test.

## RESULTS

From the initial cohort of 48 902 patients [8], 8649 had microbiological investigations recorded and 4092 had procalcitonin results recorded. For this analysis, we included 1040 people with both an admission procalcitonin result and microbiological investigations within 2 days of hospital admission (characteristics summarized in Supplementary Table 1). These patients had a median age of 65 years (interquartile range [IQR], 53–77 years) and 635 (61.1%) were male. Four hundred nine (39.3%) required critical care admission and 301 (29.5%) received invasive mechanical ventilation (IMV). Three hundred forty-six patients died in hospital (33.3%). Compared to the entire initial cohort, patients included in this analysis were younger, more likely to have chest radiograph infiltrates, and more likely to be admitted to critical care and receive IMV; the results of this analysis are therefore more applicable to this subgroup of patients (Supplementary Table 1).

Blood culture results alone were recorded for 946 (91%) patients, respiratory culture results alone for 58 (5.6%), and both for 36 (3.5%). Overall, 170 (17.3%) blood and 60 (63.8%) of respiratory cultures were positive. Blood and respiratory cultures from 6 patients were both positive but with different pathogens in 5 of 6 cases (in 1 case a β-hemolytic *Streptococcus* was isolated from both). As previously reported, *Staphylococcus aureus* and gram-negative bacilli were the most prevalent pathogens (Supplementary Figure 1) [8].

**Figure 1.**
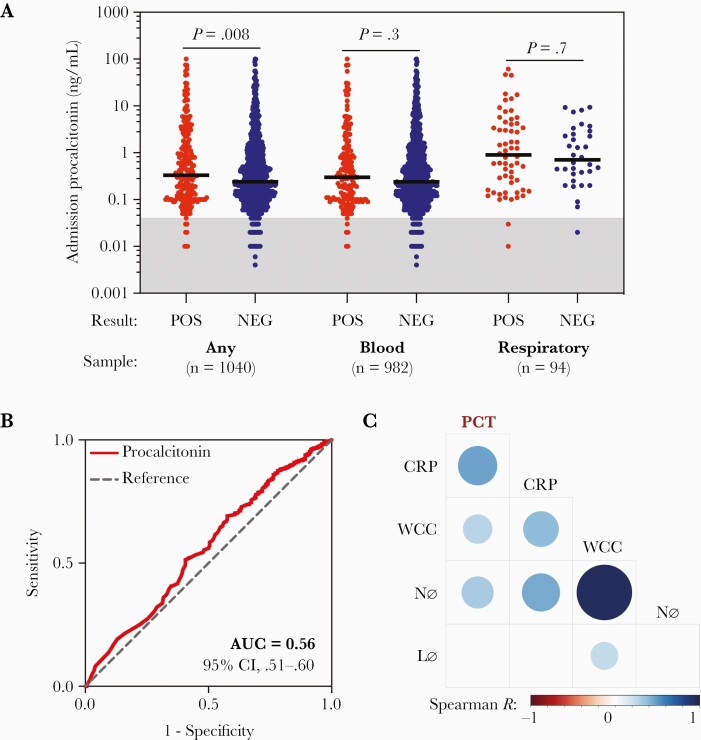
Evaluation of admission procalcitonin as a biomarker of bacterial coinfection in coronavirus disease 2019. *A*, Comparison of admission procalcitonin concentrations between patients with positive and negative microbiology results within the first 2 days of admission. “Any” refers to either blood or respiratory samples, representing the entire cohort. Lines show the median. The gray shading identifies values ≤0.04 ng/mL, which is the mean concentration measured in healthy people [9]. Groups were compared with individual Mann-Whitney tests. *P* values shown are uncorrected. *B*, Receiver operating characteristic analysis for admission procalcitonin concentration in predicting absence of coinfection (considering patients with any microbiological sample available). *C*, Correlation matrix analysis of admission procalcitonin, C-reactive protein, total white cell count, neutrophil count, and lymphocyte count. The size and shading of circles represents the Spearman *R* value with a *P* value of <.05. An empty cell indicates no statistically significant correlation between the variables. Abbreviations: AUC, area under the curve; CI, confidence interval; CRP, C-reactive protein; Lø, lymphocyte count; Nø, neutrophil count; PCT, procalcitonin; WCC, white cell count.

The median admission procalcitonin concentration for patients with any positive culture (n = 224) was 0.33 (IQR, 0.11–1.70) ng/mL compared to 0.24 (IQR, 0.10–0.90) ng/mL for negative cultures (n = 816; *P* = .008; Figure 1A). Median procalcitonin for patients with positive blood cultures (n = 170) was 0.30 (IQR, 0.10–1.11) ng/mL compared to 0.24 (IQR, 0.10–0.90) ng/mL for negative blood cultures (n = 812; *P* = .3). For patients with positive respiratory cultures (n = 60), median procalcitonin was 0.90 (IQR, 0.18–4.16) ng/mL compared to 0.71 (IQR, 0.33–2.59) ng/mL for negative respiratory cultures (n = 34; *P* = .7). Receiver operator characteristic analysis demonstrated that procalcitonin performed poorly as a diagnostic test (considering any culture result): the area under the curve for classifying absence of coinfection was 0.56 (95% confidence interval [CI], .51–.60; Figure 1B).

A procalcitonin threshold of 0.25 ng/mL is commonly used in trials of procalcitonin-guided antimicrobial usage (values in health are ≤0.04 ng/mL [9]). Observational data demonstrate that using a threshold of ≤0.25 ng/mL to advise against empiric antimicrobials in COVID-19 reduces antimicrobial usage [10]. In our cohort, patients with an admission procalcitonin <0.25 ng/mL were less likely to have a coinfection (91/502 [18.0%] vs 133/538 [24.4%]; *P* = .01), but the sensitivity and specificity of this threshold were low (59.4% [95% CI, 52.8%–65.6%] and 50.4% [46.9%–53.8%], respectively). Similar results were obtained using a threshold of 0.5 ng/mL (sensitivity, 44.2% [37.6%–50.9%]; specificity, 65.4% [62.1%–68.7%]). Since the prevalence of coinfection in this highly selected cohort might not be representative of all hospitalized people with COVID-19, we have deliberately not reported a negative predictive value as it could be misleading.

Correlation matrix analysis identified positive correlations of admission procalcitonin with CRP and total white cell and neutrophil counts (Figure 1C). This was investigated further using CRP, TNF-α, IL-6, and procalcitonin measurements from plasma samples from hospitalized people with COVID-19 in the ISARIC4C study [7]. Results from the same sample were available for procalcitonin and CRP (n = 94), TNF-α (n = 59), or IL-6 (n = 71). No results of microbiological investigations were recorded for these patients. This identified weak-moderate positive correlations of procalcitonin with CRP (*r* = 0.54, *P* < .0001), TNF-α (*r* = 0.36, *P* = .006), and IL-6 (*r* = 0.38, *P* = .001; Supplementary Figure 2*A–C*).

## DISCUSSION

Among hospitalized people with COVID-19 undergoing microbiological investigation for suspected bacterial coinfection (within 2 days of admission), admission procalcitonin did not reliably identify people with positive microbiological findings. Low concentrations were observed in some people with coinfection, and high concentrations in some people without. Positive correlations were identified between procalcitonin and inflammatory markers including IL-6.

Procalcitonin is elevated in COVID-19 proportional to disease severity and we propose that IL-6 mediates this independent of bacterial coinfection. In support of this, administration of recombinant IL-6 to humans and stimulation of ex vivo liver slices with IL-6 induces procalcitonin production [11]. In critically ill people with COVID-19, tocilizumab administration is associated with a blunted procalcitonin response to late-onset secondary infections [12]. In a previous study of severe influenza, we found that whole blood transcriptomic signatures characteristic of bacterial infection develop in some patients with severe disease in the second week of illness, associated with elevated procalcitonin levels without bacterial infection [13]. Severe influenza is also associated with elevated IL-6, at concentrations equivalent to COVID-19 [7, 13]. Observational clinical data from hospitalized people with respiratory virus infections prior to the COVID-19 pandemic (50% with influenza) demonstrates that procalcitonin elevation occurs in pure viral infections, is associated with disease severity, and performs poorly as a diagnostic test for bacterial coinfection [14]. Procalcitonin is also elevated and associated with severity in dengue [15] and Crimean-Congo hemorrhagic fever [16]. Overall, we conclude that procalcitonin may not be a good marker of bacterial infection in viral diseases of sufficient severity to cause hospital admission.

Our retrospective analysis has important limitations. This is a highly selected cohort derived by necessity to address our specific research question: 1040 of 48 902 (2%) patients in the cohort met inclusion criteria. Selection bias will be present, in particular (1) the degree of clinical suspicion for bacterial coinfection leading to procalcitonin and cultures being performed, (2) ability to obtain microbiological samples, and (3) intersite variability in procalcitonin usage. Rates of recorded microbiological investigation were low and culture positivity was high. There may be a bias for preferential recording of positive microbiology results in the database. Administration of antimicrobials prior to microbiological sampling in community-acquired pneumonia results in false-negative culture results when compared to bacterial PCR [17]. Clinical information regarding bacterial infection diagnosis was not available, meaning coinfection was inferred entirely from microbiology data. Finally, this analysis includes patients from the first pandemic wave in the United Kingdom. Although patterns of secondary infection may differ with subsequent usage of immunomodulators after hospitalization, these changes in practice should not influence the generalizability of our findings to the diagnosis of coinfections at the time of hospital admission. It is important to note that our findings do not relate to use of procalcitonin to diagnose secondary infections (eg, ventilator-associated pneumonia) or serial measurements to observe trends over time in relation to development of secondary infections, where procalcitonin may have greater utility [18].

In conclusion, our study of procalcitonin level at admission to hospital in people with COVID-19 being investigated for suspected bacterial coinfection showed that procalcitonin was not a reliable marker for positive microbiological investigations. Overall, procalcitonin may not be a reliable indicator of bacterial infection in severe viral diseases with raised IL-6 levels. Microbiological investigation remains critical to identify coinfections and inform antimicrobial decision-making.

## Supplementary Material

ofac179_suppl_Supplementary_Material

## Notes

***Author contributions.*** C. D. R., A. H., L. T., P. J. M. O., J. K. B., and M. G. S. conceived the study. A. H., C. D. R., L. T., R. P., L. M., S. C. M., A. B. D., R. S. T., S. A., and E. M. H. curated the data. K. A. R., C. D. R., and C. J. F. did the formal analysis. P. J. M. O., J. K. B., and M. G. S. acquired the funding. H. E. H. and W. O. were project administrators. C. D. R., A. H., and M. G. S. supervised the study. K. A. R. and C. D. R. wrote the original draft of the manuscript. A. H., L. T., C. J. F., T. I. d. S., M. K. S., T. M. D., E. M. H., A. B. D., P. J. M. O., J. K. B., and M. G. S. reviewed and edited the manuscript.

***Acknowledgments.*** This work uses data provided by patients and collected by the National Health Service (NHS) as part of their care and support. We are extremely grateful to the front-line NHS clinical and research staff and volunteer medical students who collected this data in challenging circumstances; and the generosity of the participants and their families for their individual contributions in these difficult times. We also acknowledge the support of Jeremy J. Farrar (Wellcome Trust) and Nahoko Shindo (World Health Organization [WHO]).

***Data sharing.*** Access to all data and samples collected by ISARIC4C are controlled by an Independent Data and Materials Access Committee composed of representatives of research funders, academia, clinical medicine, public health, and industry. The application process for access to the data is available on the ISARIC4C website (https://isaric4c.net).

***Patient consent.*** Consent was not required for collection of depersonalized routine healthcare data for research in England and Wales. A waiver for consent was given by the Public Benefit and Privacy Panel in Scotland. Consent by, or assent for, participants was obtained for additional biological sampling for research purposes

***Financial support.*** This work was supported by the National Institute for Health Research (NIHR) (grant number CO-CIN-01); the Medical Research Council (grant number MC_PC_19059); and the NIHR Health Protection Research Unit (HPRU) in Emerging and Zoonotic Infections at University of Liverpool in partnership with Public Health England (PHE), in collaboration with Liverpool School of Tropical Medicine and the University of Oxford (grant number 200907); NIHR HPRU in Respiratory Infections at Imperial College London with PHE (grant number 200927); Wellcome Trust and Department for International Development (grant number 215091/Z/18/Z); the Bill and Melinda Gates Foundation (grant number OPP1209135); Liverpool Experimental Cancer Medicine Centre (grant number C18616/A25153); NIHR Biomedical Research Centre at Imperial College London (grant number IS-BRC-1215-20013); EU Platform for European Preparedness Against (Re-) emerging Epidemics (FP7 project 602525); and NIHR Clinical Research Network for providing infrastructure support for this research. L. T. is supported by a Wellcome Trust fellowship (grant number 205228/Z/16/Z). P. J. M. O. is supported by an NIHR Senior Investigator Award (grant number 201385).

***Potential conflicts of interest.*** All authors: No reported conflicts of interest.

## APPENDIX

ISARIC4C Investigators

*Consortium Lead Investigator*: J. Kenneth Baillie. *Chief Investigator*: Malcolm G. Semple. *Co-Lead Investigator*: Peter J. M. Openshaw. *ISARIC Clinical Coordinator*: Gail Carson. *Co-Investigator*: Beatrice Alex, Benjamin Bach, Wendy S. Barclay, Debby Bogaert, Meera Chand, Graham S. Cooke, Annemarie B. Docherty, Jake Dunning, Ana da Silva Filipe, Tom Fletcher, Christopher A. Green, Ewen M. Harrison, Julian A. Hiscox, Antonia Ying Wai Ho, Peter W. Horby, Samreen Ijaz, Saye Khoo, Paul Klenerman, Andrew Law, Wei Shen Lim, Alexander J. Mentzer, Laura Merson, Alison M. Meynert, Mahdad Noursadeghi, Shona C Moore, Massimo Palmarini, William A. Paxton, Georgios Pollakis, Nicholas Price, Andrew Rambaut, David L. Robertson, Clark D. Russell, Vanessa Sancho-Shimizu, Janet T. Scott, Thushan de Silva, Louise Sigfrid, Tom Solomon, Shiranee Sriskandan, David Stuart, Charlotte Summers, Richard S. Tedder, Emma C. Thomson, A. A. Roger Thompson, Ryan S. Thwaites, Lance C. W. Turtle, Rishi K. Gupta, Maria Zambon. *Project Manager*: Hayley Hardwick, Chloe Donohue, Ruth Lyons, Fiona Griffiths, Wilna Oosthuyzen. *Data Analyst*: Lisa Norman, Riinu Pius, Thomas M. Drake, Cameron J. Fairfield, Stephen R. Knight, Kenneth A. Mclean, Derek Murphy, Catherine A. Shaw. *Data and Information System Manager*: Jo Dalton, Michelle Girvan, Egle Saviciute, Stephanie Roberts, Janet Harrison, Laura Marsh, Marie Connor, Sophie Halpin, Clare Jackson, Carrol Gamble. *Data Integration and Presentation*: Gary Leeming, Andrew Law, Murray Wham, Sara Clohisey, Ross Hendry, James Scott-Brown. *Material Management*: William Greenhalf, Victoria Shaw, Sara McDonald. *Patient Engagement*: Seán Keating. *Outbreak Laboratory Staff and Volunteers*: Katie A. Ahmed, Jane A. Armstrong, Milton Ashworth, Innocent G. Asiimwe, Siddharth Bakshi, Samantha L. Barlow, Laura Booth, Benjamin Brennan, Katie Bullock, Benjamin W. A. Catterall, Jordan J. Clark, Emily A. Clarke, Sarah Cole, Louise Cooper, Helen Cox, Christopher Davis, Oslem Dincarslan, Chris Dunn, Philip Dyer, Angela Elliott, Anthony Evans, Lorna Finch, Lewis W. S. Fisher, Terry Foster, Isabel Garcia-Dorival, William Greenhalf, Philip Gunning, Catherine Hartley, Rebecca L. Jensen, Christopher B. Jones, Trevor R. Jones, Shadia Khandaker, Katharine King, Robyn T. Kiy, Chrysa Koukorava, Annette Lake, Suzannah Lant, Diane Latawiec, Lara Lavelle-Langham, Daniella Lefteri, Lauren Lett, Lucia A. Livoti, Maria Mancini, Sarah McDonald, Laurence McEvoy, John McLauchlan, Soeren Metelmann, Nahida S. Miah, Joanna Middleton, Joyce Mitchell, Shona C. Moore, Ellen G. Murphy, Rebekah Penrice-Randal, Jack Pilgrim, Tessa Prince, Will Reynolds, P. Matthew Ridley, Debby Sales, Victoria E. Shaw, Rebecca K. Shears, Benjamin Small, Krishanthi S. Subramaniam, Agnieska Szemiel, Aislynn Taggart, Jolanta Tanianis-Hughes, Jordan Thomas, Erwan Trochu, Libby van Tonder, Eve Wilcock, J. Eunice Zhang, Lisa Flaherty, Nicole Maziere, Emily Cass, Alejandra Doce Carracedo, Nicola Carlucci, Anthony Holmes, Hannah Massey. *Edinburgh Laboratory Staff and Volunteers*: Lee Murphy, Nicola Wrobel, Sarah McCafferty, Kirstie Morrice, Alan MacLean. *Local Principal Investigators*: Kayode Adeniji, Daniel Agranoff, Ken Agwuh, Dhiraj Ail, Erin L. Aldera, Ana Alegria, Brian Angus, Abdul Ashish, Dougal Atkinson, Shahedal Bari, Gavin Barlow, Stella Barnass, Nicholas Barrett, Christopher Bassford, Sneha Basude, David Baxter, Michael Beadsworth, Jolanta Bernatoniene, John Berridge, Nicola Best, Pieter Bothma, David Chadwick, Robin Brittain-Long, Naomi Bulteel, Tom Burden, Andrew Burtenshaw, Vikki Caruth, David Chadwick, Duncan Chambler, Nigel Chee, Jenny Child, Srikanth Chukkambotla, Tom Clark, Paul Collini, Catherine Cosgrove, Jason Cupitt, Maria-Teresa Cutino-Moguel, Paul Dark, Chris Dawson, Samir Dervisevic, Phil Donnison, Sam Douthwaite, Ingrid DuRand, Ahilanadan Dushianthan, Tristan Dyer, Cariad Evans, Chi Eziefula, Christopher Fegan, Adam Finn, Duncan Fullerton, Sanjeev Garg, Sanjeev Garg, Atul Garg, Effrossyni Gkrania-Klotsas, Jo Godden, Arthur Goldsmith, Clive Graham, Elaine Hardy, Stuart Hartshorn, Daniel Harvey, Peter Havalda, Daniel B. Hawcutt, Maria Hobrok, Luke Hodgson, Anil Hormis, Michael Jacobs, Susan Jain, Paul Jennings, Agilan Kaliappan, Vidya Kasipandian, Stephen Kegg, Michael Kelsey, Jason Kendall, Caroline Kerrison, Ian Kerslake, Oliver Koch, Gouri Koduri, George Koshy, Shondipon Laha, Steven Laird, Susan Larkin, Tamas Leiner, Patrick Lillie, James Limb, Vanessa Linnett, Jeff Little, Mark Lyttle, Michael MacMahon, Emily MacNaughton, Ravish Mankregod, Huw Masson, Elijah Matovu, Katherine McCullough, Ruth McEwen, Manjula Meda, Gary Mills, Jane Minton, Mariyam Mirfenderesky, Kavya Mohandas, Quen Mok, James Moon, Elinoor Moore, Patrick Morgan, Craig Morris, Katherine Mortimore, Samuel Moses, Mbiye Mpenge, Rohinton Mulla, Michael Murphy, Megan Nagel, Thapas Nagarajan, Mark Nelson, Matthew K. O’Shea, Igor Otahal, Marlies Ostermann, Mark Pais, Selva Panchatsharam, Danai Papakonstantinou, Hassan Paraiso, Brij Patel, Natalie Pattison, Justin Pepperell, Mark Peters, Mandeep Phull, Stefania Pintus, Jagtur Singh Pooni, Frank Post, David Price, Rachel Prout, Nikolas Rae, Henrik Reschreiter, Tim Reynolds, Neil Richardson, Mark Roberts, Devender Roberts, Alistair Rose, Guy Rousseau, Brendan Ryan, Taranprit Saluja, Aarti Shah, Prad Shanmuga, Anil Sharma, Anna Shawcross, Jeremy Sizer, Manu Shankar-Hari, Richard Smith, Catherine Snelson, Nick Spittle, Nikki Staines, Tom Stambach, Richard Stewart, Pradeep Subudhi, Tamas Szakmany, Kate Tatham, Jo Thomas, Chris Thompson, Robert Thompson, Ascanio Tridente, Darell Tupper-Carey, Mary Twagira, Andrew Ustianowski, Nick Vallotton, Lisa Vincent-Smith, Shico Visuvanathan, Alan Vuylsteke, Sam Waddy, Rachel Wake, Andrew Walden, Ingeborg Welters, Tony Whitehouse, Paul Whittaker, Ashley Whittington, Padmasayee Papineni, Meme Wijesinghe, Martin Williams, Lawrence Wilson, Sarah Cole, Stephen Winchester, Martin Wiselka, Adam Wolverson, Daniel G. Wootton, Andrew Workman, Bryan Yates, Peter Young.
