# Supplementary material for: Procalcitonin Is Not a Reliable Biomarker of Bacterial Coinfection in People With Coronavirus Disease 2019 Undergoing Microbiological Investigation at the Time of Hospital Admission
Source: Open Forum Infect Dis. 2022 May 1;9(5):ofac179. doi: 10.1093/ofid/ofac179 (PMC9070482; doi:10.1093/ofid/ofac179)
Supplement: ofac179_suppl_Supplementary_Material [file ofac179_suppl_supplementary_material.pdf]

Procalcitonin is not a reliable biomarker of bacterial co-infection in COVID-19 at the time of hospital admission

Supplementary Material

Contents:

Supplementary Table 1: Characteristics of 1040 included patients.. 2

Supplementary Figure 1: Microbiological findings 3

Supplementary Figure 2: Relationship of plasma procalcitonin with CRP, IL-6 and TNF-α.................. 4

Supplementary Table 1: Characteristics of 1040 included patients

Variable	n[a]	Included patients (n=1040)		Entire cohort (n=48,902)
**Patient characteristics**				
Male sex, n (%)	1039	635	(61.1%)	57.4%
Age, years	1037	65	(53-77)	74 (59-84)
Co-morbidities, n (%)				
Hypertension	652	326	(50.0%)	48.4%
Chronic lung disease	1014	301	(29.7%)	28.7%
Diabetes mellitus	1015	265	(26.1%)	29.4%
Chronic cardiac disease	1000	259	(25.9%)	32.3%
Immunocompromise	713	162	(22.7%)	29.3%
Obesity	889	196	(22.0%)	11.9%
Chronic kidney disease	1002	150	(15.0%)	17.8%
Liver disease	993	21	(2.1%)	1.9%
**Physiological parameters**[b]				
Temperature, °C	1029	37.6	(36.8-38.4)	37.4 (36.3-38.5)
Respiratory rate, breaths/min	1029	24	(20-30)	21 (18-26)
Systolic blood pressure, mmHg	1028	129	(114-145)	131 (106-156)
Heart rate, beats/min	1033	94	(83-109)	92 (71-113)
SpO2, %	1026	94	(90-96)	95 (92-97)
**Investigations**[b]				
Chest x-ray infiltrates, n (%)[c]	850	650	(76.5)	67.5%
Total white cell count, x10^9/L	1035	7.5	(5.5-10.5)	7.3 (5.3-10.3)
C-reactive protein, mg/L	1028	116	(56-196)	83 (35-157)
Procalcitonin, ng/mL	1040	0.28	(0.1-1.0)	0.3 (0.1-1.1)
**Supportive care**, n (%)				
Critical care admission	1040	409	(39.3%)	14.5%
Invasive mechanical ventilation	1019	301	(29.5%)	9.3%
**Outcomes**, n (%)	1040			
Discharged alive		591	(56.8%)	58.9%
In-hospital mortality		346	(33.3%)	31.5%
Transfer to another facility		52	(5.0%)	5.4%
Ongoing hospitalisation		43	(4.1%)	2.4%
Palliative discharge		8	(0.8%)	1.8%

Data are median (IQR) unless otherwise stated.

[a]Number of patients included in procalcitonin analysis with data available

[b]Recorded on admission to hospital

[c]As a proportion of patients who had chest x-ray results recorded

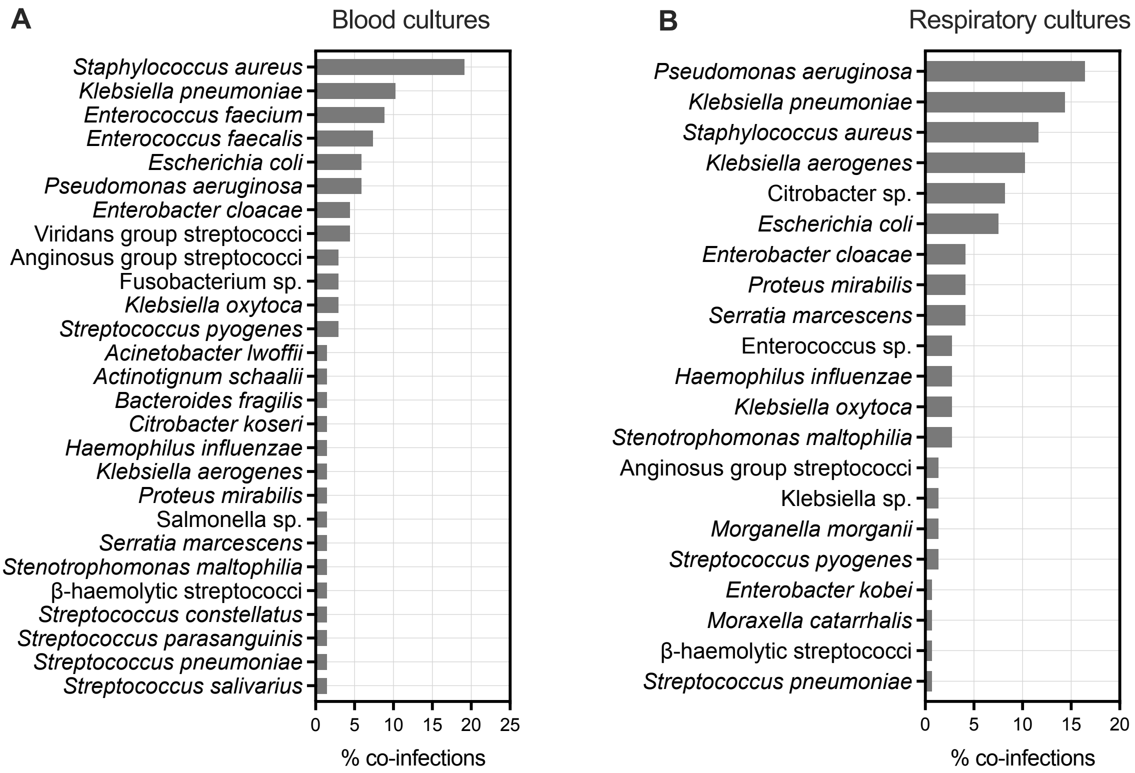

Supplementary Figure 1: Microbiological findings

Bacterial pathogens recovered from **(A)** blood and **(B)** respiratory cultures, expressed as the percentage of co-infections from each sample type accounted for by each organism.

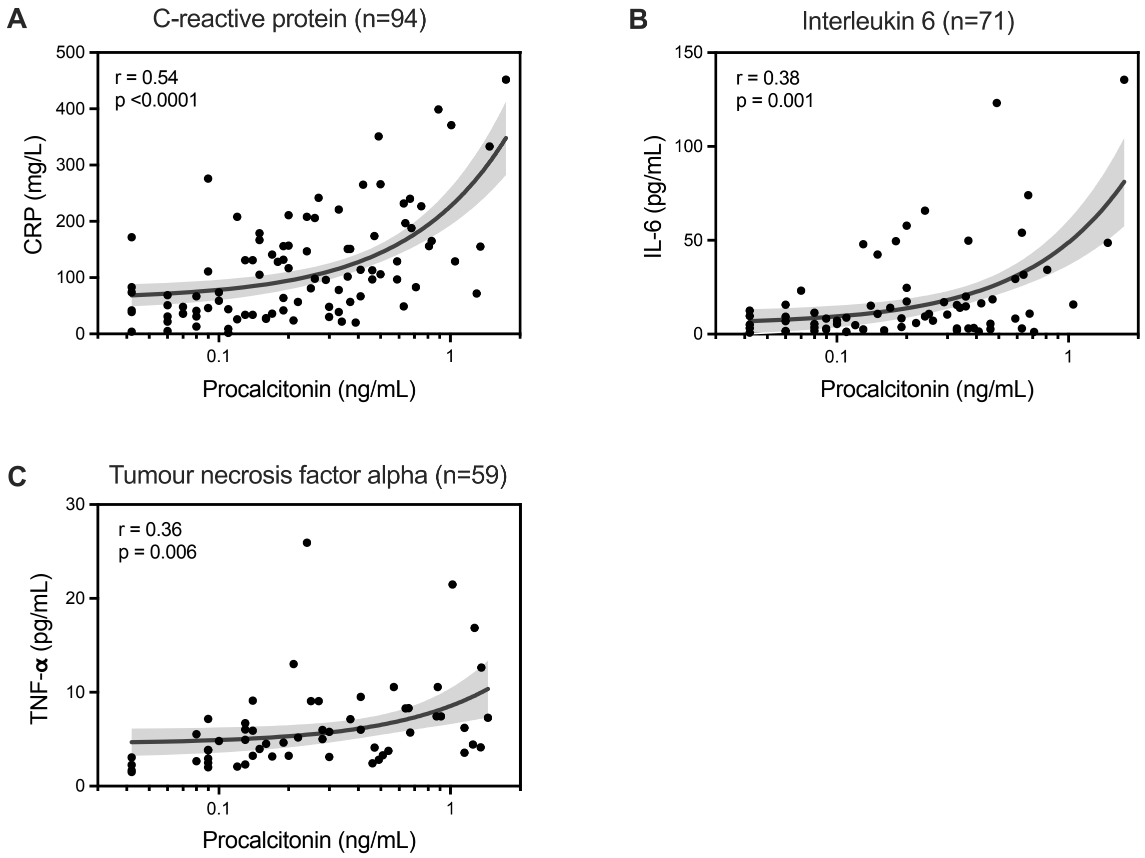

Supplementary Figure 2: Relationship of plasma procalcitonin with CRP, IL-6 and TNF-α

The plots show linear regressions with 95% confidence intervals for procalcitonin with **(A)** CRP (n=94), **(B)** IL-6 (n=71) and **(C)** TNF-α (n=59). Correlation assessed with Spearman's test. Procalcitonin measurements below the lower limit of detection (LLOD) of the assay (0.06 ng/mL) were imputed with the LLOD divided by the square root of two. The x-axis shows a Log10 scale.
